# miRNA profiling of chicken follicles during follicular development

**DOI:** 10.1038/s41598-024-52716-x

**Published:** 2024-01-26

**Authors:** Zhongxian Xu, Qian Liu, Chunyou Ning, Maosen Yang, Qing Zhu, Diyan Li, Tao Wang, Feng Li

**Affiliations:** 1 Key Laboratory of Southwest China Wildlife Resources Conservation (Ministry of Education), China West Normal University, Nanchong, 637009 China; 2 Institute of Animal Genetics and Breeding, College of Animal Science and Technology, Sichuan Agricultural University, Chengdu, 611130 China; 3 Antibiotics Research and Re-evaluation Key Laboratory of Sichuan Province, Sichuan Industrial Institute of Antibiotics, School of Pharmacy, Chengdu University, Chengdu, 610106 China

**Keywords:** Agricultural genetics, Non-coding RNAs

## Abstract

MicroRNAs (miRNAs) play a crucial role as transcription regulators in various aspects of follicular development, including steroidogenesis, ovulation, apoptosis, and gene regulation in poultry. However, there is a paucity of studies examining the specific impact of miRNAs on ovarian granulosa cells (GCs) across multiple grades in laying hens. Consequently, this study aims to investigate the roles of miRNAs in chicken GCs. By constructing miRNA expression profiles of GCs at 10 different time points, encompassing 4 pre-hierarchical, 5 preovulatory, and 1 postovulatory follicles stage, we identified highly expressed miRNAs involved in GC differentiation (miR-148a-3p, miR-143-3p), apoptosis (let7 family, miR-363-3p, miR-30c-5p, etc.), and autophagy (miR-128-3p, miR-21-5p). Furthermore, we discovered 48 developmentally dynamic miRNAs (DDMs) that target 295 dynamic differentially expressed genes (DDGs) associated with follicular development and selection (such as oocyte meiosis, progesterone-mediated oocyte maturation, Wnt signaling pathway, TGF-*β* signaling pathway) as well as follicular regression (including autophagy and cellular senescence). These findings contribute to a more comprehensive understanding of the intricate mechanisms underlying follicle recruitment, selection, and degeneration, aiming to enhance poultry’s reproductive capacity.

## Introduction

The ovary of sexually mature domestic hens (*Gallus domesticus*) exhibits a complex arrangement of follicles comprising cortical follicles, slow-growing white and yellow follicles (pre-hierarchical follicles), rapidly growing large hierarchical follicles (preovulatory follicles), and postovulatory follicles (POFs) protruding from the ovarian surface^1,2^. The continuous recruitment of small yellow follicles (SYFs) from a pool of white follicles ensures the maintenance of the SYF population. Pre-hierarchical yellowish follicles form a cohort from which a single follicle is recruited daily or near-daily into the fast-growing group of yellow hierarchical follicles^3^, with follicular atresia commonly occurring during this process. Within the preovulatory hierarchy, a single follicle is selected from a cohort of pre-hierarchical follicles, and these follicles are arranged in a well-defined hierarchical order^4^. Out of the selected preovulatory follicles (usually four to six), one matures to become the largest follicle (F1) that ovulates approximately once a day^5,6^. Following ovulation, the ruptured follicle, including the granulosa layers, forms a postovulatory follicle (POF)^7^. An intact follicle comprises oocytes, granulosa cells (GCs), and theca cells (TCs), with GCs playing a significant role in follicular development by secreting various factors and steroidal hormones^5,8^.

miRNAs are small endogenous non-coding RNAs that play a critical role in the posttranscriptional regulation of gene expression^9^. The egg-laying performance of hens is tightly regulated by the interaction between multiple miRNAs and their target genes, which modulate avian follicular development, recruitment, maturation, selection, atresia, and regression^10–13^. While many studies have focused on the transcriptional expression of miRNAs, evidence supports the idea that miRNAs primarily affect ovarian function through their actions on ovarian granulosa cells^6,14^. However, few studies have investigated the effects of miRNAs on ovarian granulosa cells at different stages in laying hens. This study aims to investigate the miRNA transcriptome profiling in GCs from peak laying hens across 10 stages of folliculogenesis (including pre-hierarchical, preovulatory, and postovulatory follicles) using high-throughput small RNA sequencing. By employing the MasigPro method, a set of developmentally dynamic miRNAs (DDMs) with similar temporal expression patterns were identified and visualized. Co-expression networks were constructed to explore the interactions between mRNA and miRNA pairs associated with the regulation of ovarian function. These findings provide a more detailed understanding of the mechanisms underlying follicle recruitment, selection, and degeneration, ultimately contributing to efforts to improve poultry's reproductive capacity.

## Materials and methods

### Ethics statement

All animal protocols conducted in this study were approved by the Institutional Animal Care and Use Committee of Sichuan Agricultural University (protocol number B20171910). The methods employed in this research were performed in strict adherence to the approved guidelines. And this study is reported in accordance with ARRIVE guidelines (https://arriveguidelines.org).

### Animals and separation of follicles

All the Luhua chickens used in this study were sourced from the Poultry Breeding Farm of Sichuan Agricultural University (Ya’an, Sichuan, China). The experimental hens were individually housed in cages within a controlled environment, a photoperiod of 14 h of light and 10 h of darkness. They were provided with *ad libitum* access to feed and water, following standard farm husbandry practices.

A total of six healthy Luhua hens at the peak egg-laying age (31 weeks old) were humanely euthanized using intravenous injection of 2% pentobarbital sodium (25 mg/kg of body weight). After opening the abdomen, the ovaries were immediately collected. Various follicles at different stages of development were carefully dissected. A total of nine levels of pre-hierarchical and preovulatory follicles were categorized based on their diameter, number, and color. Additionally, the atretic follicles, also known as postovulatory follicles (POF), which undergo rapid metabolic activity termination and regression within a few days after ovulating the most mature follicle (F1) for egg formation, were isolated. The theca and granulosa cell (GC) layers were separated using methods described in previous studies^15,16^. The GC layers were washed with PBS, flash-frozen in liquid nitrogen, and stored at − 80 °C for RNA isolation.

### Small RNA sequencing and data analysis

Total RNA was extracted from all collected samples using RNAiso Plus reagent (TaKaRa, Japan, #9108), following the manufacturer’s instructions. The integrity and quality of the total RNA were assessed using a Bioanalyzer 2100 system (Agilent Technologies, Palo Alto, CA, USA) and an RNA 6000 Nano kit. Three small RNA libraries were constructed for each stage using the QIAseq miRNA Library Kit, following the manufacturer’s protocols. The prepared libraries were sequenced on a HiSeq 2500 platform, with a single-end sequencing length of 50 bp, at Novogene Bioinformatics Technology Co., Ltd (Beijing, China).

To obtain high-quality reads, cutadapt-1.2.1 was used to filter low-quality reads and remove reads with adaptor contamination. The remaining high-quality reads were then mapped to the chicken reference genome (GRCg6a) using Bowtie. The reads that did not align to rRNA, tRNA, snRNA, scRNA, and snoRNA were aligned to miRbase to identify known miRNAs in *Gallus gallus*. The expression levels of miRs were estimated using TPM (transcripts per million). The normalization and identification of differentially expressed miRNAs (DEMs) were calculated using the EdgeR package in RStudio. DEMs with |log FC| > 1 and FDR < 0.05 were considered significant. Furthermore, maSigPro^17^ was used to identify miRNAs with significant temporal expression changes across the 10 time points developmentally dynamic miRNAs (DDMs) were defined as DEMs with a goodness-of-fit (*R*^2^) of at least 0.5.

### Target prediction and functional enrichment

The target prediction and construction of the miRNA-mRNA interaction network construction were detailed in our previous study. In brief, we employed two algorithms, TargetScan (http://www.targetscan.org/vert_61/) and miRDB (http://mirdb.org/)^18^, to predict the potential target genes of the identified miRNAs exhibiting significant expression profile differences over time. Furthermore, we investigated the targeted regulatory relationships between miRNAs and their target differentially expressed genes by retaining miRNAs whose targets overlapped with the DEGs^15^. This analysis was conducted using Cytoscape^19^. For the biological pathway enrichment analysis of DEGs in the four identified clusters, we utilized g:Profiler (https://biit.cs.ut.ee/gprofiler/gost?tdsourcetag=s_pcqq_aiomsg) with default parameters. The organism option selected was *Gallus gallus*, and the analysis was run as a multi-query. KEGG enrichment analyses of DDGs for the top expressed miRNAs, and visualization of pathways were performed using OmicShare tools, a free online platform for data analysis (https://www.omicshare.com/tools).

## Results

### Morphological characterization during chicken folliculogenesis

**Figure 1:**
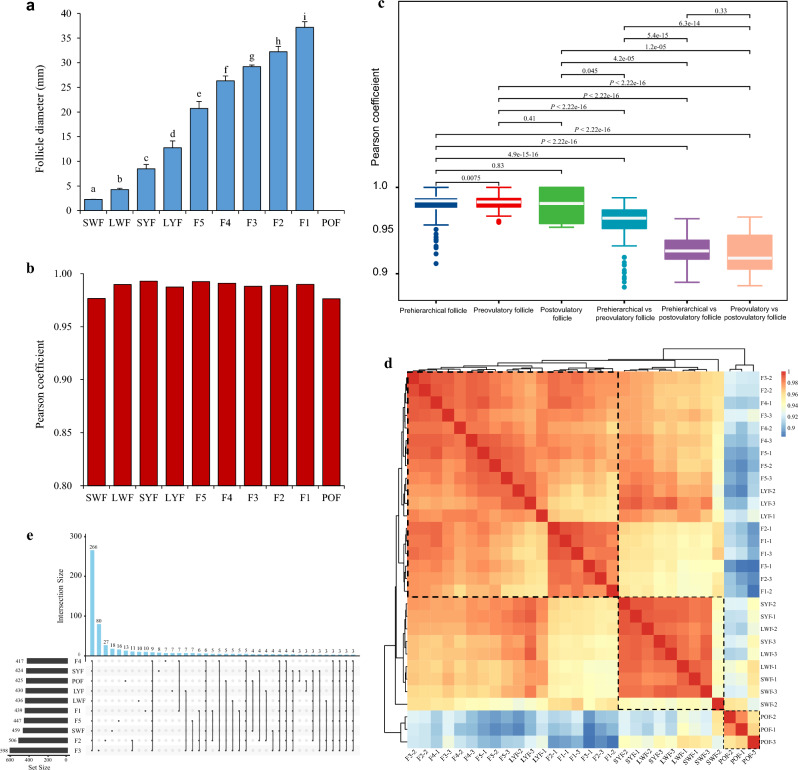
Expression profiles of miRNAs across 10 grades of follicles. (**a**) Morphological characterizations of ovarian follicles at different stages. (**b**) The Pearson correlation coefficient shows the repeatability within each sample. (**c**) Pearson correlation coefficient comparing the grades of follicles (pre-hierarchical, preovulatory, and postovulatory follicle) within and between groups. (**d**) Hierarchical clustering analysis of miRNA expression patterns. (**e**) Statistics on the number of miRNAs expressed at each stage.

Folliculogenesis refers to the progressive growth and development of ovarian follicles, which includes pre-hierarchical and hierarchical follicles leading up to ovulation or the development of atretic follicles. In our study, we classified the follicular hierarchy based on the color, number, and diameter of ovarian follicles during the peak laying period. Notably, there were significant differences in follicle diameter between adjacent developmental stages (Fig. 1a). During the pre-hierarchical stage, numerous small white follicles (SWF) and large white follicles (LWF) with diameters less than 5 mm were observed, along with 5–6 small yellow follicles (SYF) and large yellow follicles (LYF) ranging from approximately 5–12 mm in diameter. These follicles were categorized as pre-hierarchical follicles. The hierarchical stage consisted of a series of yellow yolky follicles. These preovulatory follicles were characterized by size, with diameters exceeding 20mm. They were denoted as F5 to F1, representing an order of development. During the process of follicle selection, the SYF gradually developed into preovulatory ovarian follicles, progressing from F5 to F1 until ovulation occurred. Additionally, one to two regressing POFs could be observed in the subsequent days.

### miRNA expression profiling patterns during chicken folliculogenesis

We performed a temporal small RNA analysis on chicken GCs collected at 10 stages during folliculogenesis, comprising 4 pre-hierarchical follicles, 5 preovulatory follicles, and one postovulatory follicle. A total of 413.27 million reads (M) of high-quality reads were obtained from thirty small RNA sequencing libraries, with an average depth of 13.78 M per sample. The average GC content across all samples was 48.21%. Furthermore, at least 99.12% of the reads met or exceeded Q20 (Table S1), indicating the high quality of the sequencing data. The high repeatability of each biological replicate was also confirmed by the high Pearson coefficient (Pearson’s *R* > 0.97) within samples per stage (Fig. 1b). Additionally, we observed stronger correlations within groups compared to between groups (Fig. 1c).

Hierarchical clustering analysis revealed consistent patterns between miRNA expression and morphological characteristics across the 10 developmental stages of follicle selection. The progressive phases of ovarian follicles were generally classified into two clusters: pre-hierarchical follicles (enclosed within the larger dotted box) and hierarchical follicles (enclosed within the second largest dotted box). The POFs are distinct from the other stages (enclosed within the smallest dotted box) (Fig. 1d). Interestingly, in our study, the LYFs (also referred to as F6, the smallest preovulatory follicle) appeared to be mixed with F5. We identified 834 known chicken miRNAs expressed in at least one of the samples by aligning against the miRBase database. Among them, approximately 32% were expressed across all 10 stages, with the majority (598) expressed in F3, followed by F2, SWF, and F5 (Fig. 1e).

### Predominantly expressed miRNAs in chicken GCs

**Figure 2:**
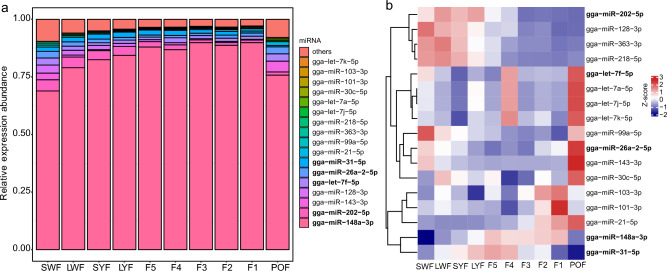
Expression dominance of the top 10 miRNAs in chicken GCs. (**a**) Relative expression abundance of the top 10 miRNAs at each stage (**b**) Z-score normalized expression levels of the top 10 miRNAs at each stage.

Previous studies have indicated that a few highly expressed miRNAs account for most detected expression, with more than 97% of expression attributed to the top 10 expressed miRNAs at each stage. Among the top 10 expressed miRNAs across the 10 stages, only 17 miRNAs were common. Five of the most abundant miRNAs, namely gga-miR-148a-3p, gga-miR-202-5p, gga-let-7f-5p, gga-miR-26a-2-5p, and gga-miR-31-5p were co-expressed in all ten stages (Fig. 2a and Table 1). Notably, gga-148a-3p, gga-miR-30a-5p, gga-miR-30a-3p, gga-miR-30b-5p, and gga-miR-218-5p were the most highly expressed miRNAs between broody and laying chicken GCs^20^.

gga-miR-148a-3p exhibited the highest abundance across the 10 stages, with its relative expression accounting for 84.60% of the total miRNA expression. Its expression increased with developmental stages from SWF to F1, peaked at F1, and declined at POF. Similar expression patterns were observed for gga-miR-31-5p (Fig. 2 and Table 1). These findings suggest that these miRNAs may play crucial roles in follicular development, particularly during follicle selection and ovulation. gga-miR-202-5p, gga-miR-128-3p, gga-miR-363-3p, and gga-miR-218-5p were found to be overexpressed in pre-hierarchical follicles, with their expression levels exhibiting a continuous decline after follicle selection and remaining low before and after ovulation (Fig. 2b). These expression patterns align with their down-regulation in broody chicken GCs^20^, indicating their involvement in promoting abnormal cell apoptosis during folliculogenesis. The members of the let-7 family, including gga-let-7f-5p/7a-5p/7j-5p/7k-5p, showed high expression at F4 and POF (Fig. 2b), suggesting their role in follicular atresia. gga-miR-26a-2-5p and gga-miR-21-5p exhibited an increase in expression with follicle maturation, peaking at POF (Fig. 2b), coinciding with the phase of follicular degeneration and atresia. This indicates their potential role in preventing apoptosis and increasing the ovulation rate in chicken GCs^21–23^. Furthermore, gga-miR-99a-5p and gga-miR-143-3p displayed a gradual decline in expression in pre-hierarchical follicles, followed by a sudden increase at POF (Fig. 2b), suggesting their involvement in follicle selection in hens with high reproductive performance^24^. gga-miR-30c-5p and gga-103-3p exhibited fluctuating expression levels, with high expression observed in POF, LWF, and F5 (Fig. 2b), indicating their roles in follicle recruitment, selection, and ovulation.

**Table 1:** The top 10 most highly expressed miRNAs, as well as their biological functions.

miRNA	TPM	Rate of TPM	Functions	References
gga-miR-148a-3p	5,581,958.47	84.60%	Facilitates GC differentiation	^20,25^
gga-miR-202-5p	166,591.99	2.52%	Follicle development	^3,26^
gga-miR-31-5p	115,657.86	1.75%	Follicle development	^3^
gga-let-7f.-5p	112,733.50	1.71%	Promotes GC apoptosis	^10,21–23,27,28^
gga-miR-26a-2-5p	101,476.17	1.54%	Promotes GC apoptosis	^21–23,29^
gga-miR-128-3p	74,340.05	1.13%	Induces GC autophagy	^30–32^
gga-miR-143-3p	52,742.49	0.80%	Facilitates GC differentiation	^22,24,33^
gga-miR-21-5p	35,211.29	0.53%	Inhibits apoptosis	^10,21–23,34^
gga-miR-30c-5p	25,591.13	0.39%	Promotes GC apoptosis	^20,22,35,36^
gga-miR-103-3p	23,250.33	0.35%	Aggravate the progress of PCOS	^37,38^
gga-miR-101-3p	20,822.41	0.32%	Promotes GC apoptosis	^11,39–41^
gga-miR-99a-5p	20,581.92	0.31%	Promotes GC apoptosis	^42,43^
gga-let-7a-5p	20,080.83	0.30%	Promotes GC apoptosis	^10,21–23,27,28^
gga-let-7j-5p	20,073.77	0.30%	Promotes GC apoptosis	^10,21–23,27,28^
gga-miR-363-3p	17,853.86	0.27%	Promotes GC apoptosis	^44,45^
gga-miR-218-5p	16,149.38	0.24%	Promotes GC apoptosis	^20,44,46^
gga-let-7 k-5p	15,739.35	0.24%	Promotes GC apoptosis	^10,21–23,27,28^

TPM represents the mean expression level of a single miRNA across 30 libraries, the Rate of TPM represents the proportion of a single miRNA’s mean expression level compared to all miRNAs.

### Expression dynamics of miRNAs throughout chicken folliculogenesis

**Figure 3:**
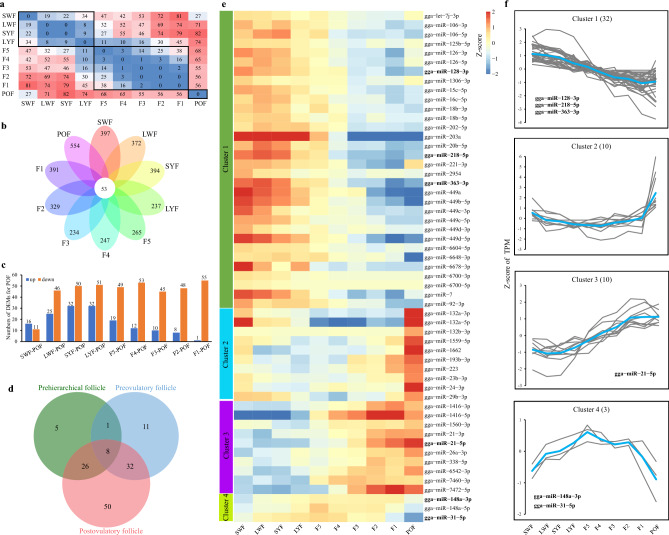
Expression dynamics of miRNAs during chicken folliculogenesis. (**a**) Comparison of DEMs through pairwise comparison at each time point. (**b**) Venn diagram showing the interactive dynamics of DEMs at each time point, with the number in the middle representing the core DEMs expressed across all time points and the numbers around the petals indicating the total number of DEMs expressed at each time point. (**c**) Histogram illustrating the distribution of up-and down-regulated DEMs in POF. (**d**) Venn diagram displaying the interactive dynamics of DEMs between different grades of follicles (pre-hierarchical, preovulatory, and postovulatory follicles), with the number in the middle representing the core DEMs expressed across the three grades and the numbers around the petals indicating the number of unique DEMs for each grade. (**e**) Heatmap showing the normalized expression levels (Z-scores of TPM) for each DDM across the four clusters. (**f**) Temporal expression profiles of the four clusters, with grey lines representing expression levels for individual DDMs, and blue lines representing the mean expression levels for all DDMs within the respective cluster during folliculogenesis.

We observed a total of 176 unique DEMs through pairwise comparisons at each time point. Comparisons between non-consecutive time points resulted in more DEMs than consecutive time points (Fig. 3a, Table S2). Among all stages, POF exhibited the highest number of DEMs (554), followed by 397 DEMs for SWF and 394 DEMs for SYF. Additionally, there were 53 core DEMs that exhibited differential expression across all time points (Fig. 3a,b). The number of DEMs within the pre-hierarchical follicle (40 DEMs), preovulatory follicle (52 DEMs), and post-preovulatory follicle (116 DEMs) periods was relatively low. Notably, the comparison between POF and SWF revealed the smallest number of DEMs, consistent with the HCL tree and Pearson correlation results. Moreover, the number of DEMs increased during the pre-hierarchical follicle period and decreased during the preovulatory follicle period, particularly among the up-regulated DEMs (Fig. 3c,d).

To investigate miRNAs associated with chicken follicular development, we identified developmentally dynamic miRNAs (DDMs) using an R package designed for transcriptomic time courses. A total of 55 miRNAs were categorized as DDMs during the egg-laying cycle (Table S2) and were divided into 4 clusters based on the regression of their expression levels at each time point. Cluster 1, characterized by a decreasing trend, consisted of the highest number of miRNAs (32 miRNAs) that exhibited high expression in the pre-hierarchical follicles and decreased during the transition from F5 to F1, including POF. Cluster 2 and Cluster 3 were the second largest clusters, each comprising 10 miRNAs. Cluster 2 (POF dominating trend) consisted of miRNAs with relatively balanced expression in both pre-hierarchical and hierarchical follicles, exhibiting a sharp increase in POF. In contrast, Cluster 3 (increasing trend) showed an increased expression pattern with developmental stages. Additionally, Cluster 4 (bell-shaped trend) consisted of 3 miRNAs (gga-miR-148a-3p, gga-miR-148a-5p, and gga-miR-31-5p) whose expression initially increased and then decreased after F5 (Fig. 3e**)**. Intriguingly, gga-miR-148a-3p and gga-miR-31-5p (Cluster 4), gga-miR-202-5p, gga-miR-128-3p, gga-miR-363-39, and gga-miR-218-5p (Cluster 1), and gga-miR-21-5p (Cluster 3) were among the top 10 highly expressed miRNAs (Fig. 3f).

### Integrative analysis of DDMs and DDGs during chicken follicle development

**Figure 4:**
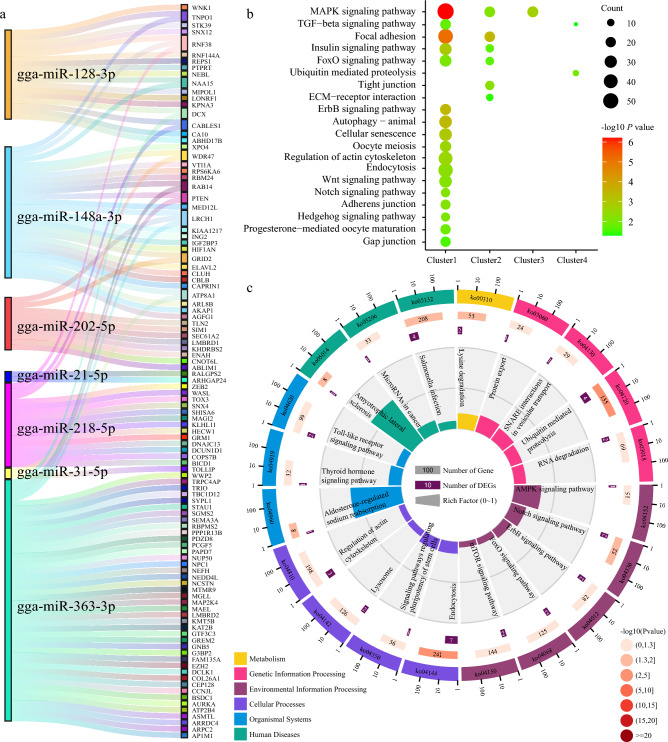
Target genes and pathways of developmentally dynamic miRNAs. (**a**) Sankey diagram showing the target genes of the top 7 highly expressed miRNAs. (**b**) Significantly enriched pathways associated with the target genes of all dynamic differentially expressed miRNAs. (**c**) Pathways enriched by the target genes of the top 10 highly expressed miRNAs.

To identify the potential targets of the 55 DDMs that changed with time-series during chicken GCs development, we employed a combination of TargetScan and miRDB to predict intersected targets. These predicted targets were then overlapped with 3669 DDGs identified in our previous study using MasigPro^15^. This is just overlapping and a simple correlation, and we did not have any evidence that these miRNAs and genes were negatively or positively correlated. Subsequently, we constructed regulatory networks between miRNA:mRNA pairs using Cytoscape. This analysis revealed significant regulatory relationships between 48 DDMs and 295 target DDGs (Table S4). Notably, gga-miR-363-3p exhibited the largest number of targets (46 DDGs), followed by gga-miR-206-55 (36 DDGs) and gga-miR-106-5p (35 DDGs). Among these 295 targets, 105 DDGs were targeted by 7 of the most highly expressed miRNAs (Fig. 4a). Specifically, gga-miR-148a-3p, which was the most highly expressed miRNA across all 10 stages, targeted 25 DDGs, including *PTEN*, *ING2*, *RNF38*, *XPO4*, *IGF2BP3*, *AGFG1*, and others.

### Key signaling pathways during chicken follicle development

To gain insights into the potential biological functions of target genes regulated by differentially expressed miRNAs with dynamic expression patterns over time, we performed functional enrichment analysis. The results revealed the enrichment of pathways associated with various stages of follicle development. For Cluster 1 (decreasing trend), which is characterized by decreasing expression over time, the target genes were enriched in pathways related to follicular development and ovulation, including the Wnt signaling pathway, TGF-β signaling pathway, Oocyte meiosis, and Progesterone-mediated oocyte maturation. Additionally, pathways associated with follicular regression, such as Autophagy and Cellular senescence, were enriched. Cluster 2 (POF dominating trend), characterized by a dominant expression in the postovulatory follicle, showed enrichment in pathways such as Tight junction and ECM-receptor interaction, which are involved in follicular development. Cluster 4 (bell-shaped trend), exhibiting an initial increase and subsequent decrease in expression, showed enrichment in the Ubiquitin mediated proteolysis pathway. Moreover, the target genes of Cluster 3, which showed an increasing trend in expression, were associated with the MAPK signaling pathway (Fig. 4b and Table S5). Furthermore, the target genes of the top 10 highly expressed miRNAs were also enriched in similar pathways. These included Endocytosis, Ubiquitin mediated proteolysis, Notch signaling pathway, and the pathways above related to follicular development, ovulation, and regression (Fig. 4c and Table S6).

**Figure 5:**
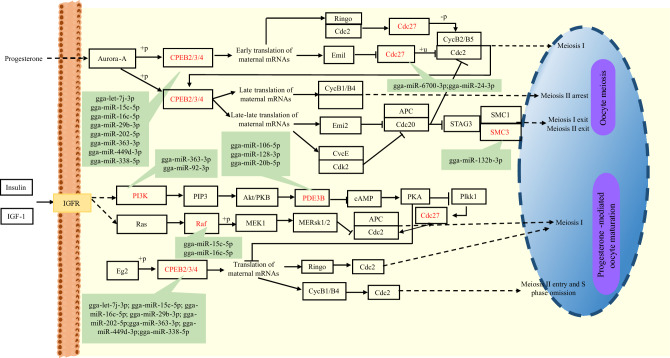
Schematic representation of miRNA-mRNA pairs and pathways related to follicular development.

The network construction of miRNA-mRNA-pathway revealed the enrichment of genes involved in oocyte meiosis, with 31 genes (corresponding to 31 miRNAs), and progesterone-mediated oocyte maturation, with 22 genes (including 28 miRNAs) (Table S7). These pathways are closely related to follicular development and maturation. Among the identified genes, *CPEB2/3/4*, targeted by 8 miRNAs including gga-let7j-3p, gga-miR-202-5p, gga-miR-363-3p, and gga-miR-92-3p, are known transcription factors involved in oogenesis and spermatogenesis. *CDC27*, targeted by gga-miR-6700-3p and gga-miR-24-3p, may play a role in regulating the timing of mitosis (Fig. 5). Notably, miR-128-3p, which has been implicated in regulating chicken GC functions through the 14-3-3β/FoxO and PPAR-γ/LPL pathways^47^, was also found to participate in oocyte meiosis (targeting: *YWHAB*, *PPP1CC*, and *MAPK14*) and progesterone-mediated oocyte maturation (targeting: *MAPK14* and *PDE3B*) (Fig. 5 and Table S7). The decreased expression of miR-20b-5p and miR-363-3p may be implicated in the occurrence and progression of human polycystic ovary syndrome (PCOS)^48^.

**Figure 6:**
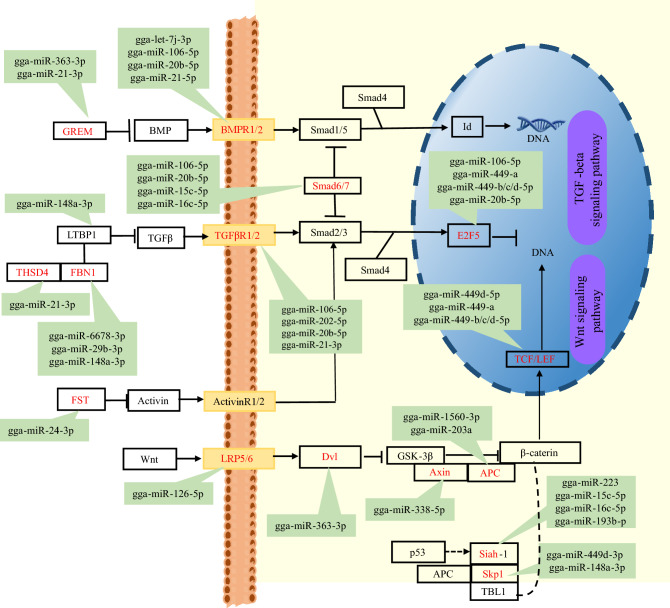
Schematic representation of miRNA-mRNA pairs and pathways involved in the follicular selection.

The TGF-beta signaling pathway (32 miRNAs: 29 target genes) and Wnt signaling pathway (32 miRNAs: 39 genes) were identified to participate in follicular selection (Table S7). Within the TGF-beta signaling pathway, SMD6/7 were recruited to the *TGFBRs* and targeted by gga-miR-106-5p, gga-miR-20b-5p, gga-miR-15c-5p, and gga-miR-16c-5p. Other miRNA-mRNA pairs such as gga-let-7j-39, gga-miR-106-5p, gga-miR-20b-5p, gga-miR-21-5p targeting *BMPR2* and gga-miR-106-5p, gga-miR-202-5p, gga-miR-20b-5p, gga-miR-21-3p targeting *TGFBR1/2* were also identified. The *GREM2* gene, which encodes BMP antagonists, was targeted by gga-miR-363-3p. The Wnt signaling pathway, closely interacting with the TGF-beta signaling pathway, was enriched by 39 genes. LRP6, targeted by gga-miR-126-5p, encodes co-receptor for Wnt and transmits the canonical Wnt signaling cascade. This pathway ultimately affects the cell cycle through *TCF* and *LEF1*, which are targeted by the gga-miR-449 family. Ubiquitin-mediated proteolysis, regulated by miRNAs gga-miR-223, gga-miR-15c-5p, gga-miR-16c-5p, gga-miR-193b-3p:SIAH1, and gg-miR-449d-3p, gga-miR-148a-3p:SKP1. Negative regulators of the Wnt signaling pathway, *Axin* (targeted by gga-miR-338-5p) and *APC* (targeted by gga-miR-203a and gga-miR-1560-3p), can induce apoptosis (Fig. 6). Knockdown of miR-21 in GCs has been shown to increase apoptosis and reduce ovulation rate^10,21–23,34^. This miRNA may regulate the TGF-beta and Wnt signaling pathways by targeting *THSD4*, EP300, *GREM2*, *TGFBR2*, and *VANGL2* (Fig. 6 and Table S7).

**Figure 7:**
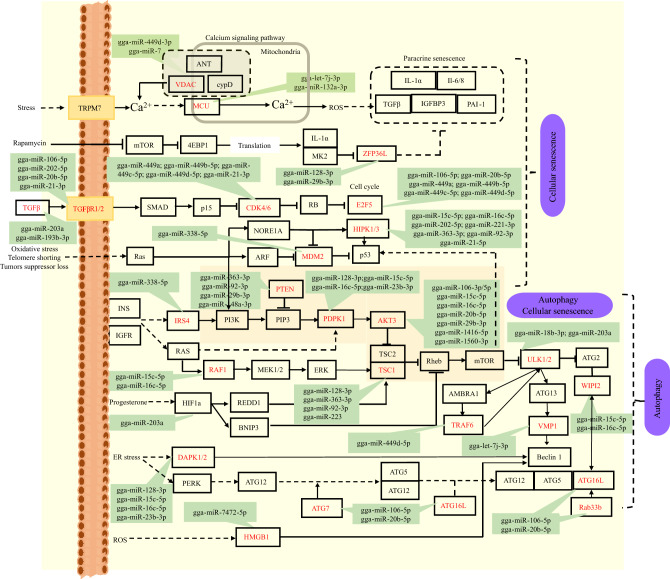
Schematic representation of miRNA-mRNA pairs and pathways associated with follicular atresia.

Follicular atresia is associated with pathways such as Autophagy (32 miRNAs: 40 targets) and Cellular senescence (36 miRNAs: 37 targets), as revealed by enrichment analysis (Table S7). *TGFB*, known for its regulatory role in cell proliferation, apoptosis, differentiation, and migration, was influenced by gga-miR-193b-3p, while *TGFBRs* were targeted by gga-miR-106-5p, gga-miR-202-5p, gga-miR-203a, gga-miR-20b-5p, and gga-miR-21-3p. Notably, regulatory pairs such as *E2F3/5/7*: gga-miR-106-5p/gga-miR-15c-5p/gga-miR-16c-5p/gga-miR-20b-5p/gga-miR-449 family and *PTEN*: gga-miR-363-3p/gga-miR-92-3p/gga-miR-29b-3p/gga-miR-148-3 were identified, emphasizing their crucial role in cell cycle control and tumor suppression. Autophagy-related genes (*ATG7* and *14*) targeted by gga-miR-106-5p, gga-miR-203a, gga-miR-20b-5p, gga-miR-363-3p, and gga-miR-92-3p were found to modulate p53-dependent cell cycle pathways and mitophagy (Fig. 7).

Pathways related to cellular adhesions play a crucial role in cell motility, proliferation, and differentiation. We identified enrichment of pathways such as Focal adhesion (40 miRNAs targeting 58 mRNAs), Adherens junction (24 miRNAs targeting 24 mRNAs), Tight junction (30 miRNAs targeting 33 mRNAs), and ECM-receptor interaction (24 miRNAs targeting 22 mRNAs), all of which are associated with follicular development (Table S7).

## Discussion

Understanding the molecular mechanisms underlying egg-laying performance in hens is crucial for improving poultry breeding strategies. In this study, we performed small RNA-seq analysis on chicken GCs collected from pre-hierarchical, preovulatory, and postovulatory follicles across 10 stages of the laying cycle. We identified a total of 834 known miRNAs, among which 17 highly expressed miRNAs associated with GC development and apoptosis accounted for a significant proportion. Differential expression analysis identified 55 developmentally dynamic miRNAs (DDMs) out of the 176 DEMs. Among these DDMs, 48 miRNAs showed significant regulatory relationships with 295 targeted differentially expressed genes. We gained insights into the functional pathways involving the identified DDMs by constructing co-expression networks of miRNA-mRNA pairs. We predicted potential miRNA-target interactions in pathways related to follicular development, selection, maturation, ovulation, and atresia. Interestingly, many miRNA-mRNA pairs we identified have been previously validated and studied in other experimental settings.

Notably, miR-128-3p, which has been implicated in regulating chicken GC functions through the 14-3-3β/FoxO and PPAR-γ/LPL pathways^47^, was also found to participate in oocyte meiosis (targeting: *YWHAB*, *PPP1CC*, and *MAPK14*) and progesterone-mediated oocyte maturation (targeting: *MAPK14* and *PDE3B*) (Fig. 5 and Table S5). The decreased expression of miR-20b-5p and miR-363-3p may be implicated in the occurrence and progression of human polycystic ovary syndrome (PCOS)^48^. miR-202-5p in geese has been shown to inhibit lipid deposition and progesterone synthesis by targeting *ACSL3* in hierarchical GCs^49^. It also regulates the proliferation and apoptosis of GCs during follicular selection by targeting *BTBD10* through the PI3KCB/AKT1 signaling pathway^50^. In chicken, miR-148a-3p upregulation in GCs facilitates steroid hormone biosynthesis by targeting *OSBP11*^20^. miR-31-5p and miR-202-5p target matrix metalloproteinase and lipid-metabolism-related genes to regulate ovarian development in hens^3^, while miR-20b-5p is associated with the proliferation of chicken primordial germ cells (PGCs)^51^. miR-26a-5p promotes theca cell proliferation by targeting *TNRC6A*^29^, and miR-99a inhibits cell proliferation by targeting *SMARCA5* in disease-infected chickens^43^. miR-143 is involved in the regulation of cell proliferation and apoptosis^33^, it negatively regulates steroid hormone synthesis and secretion, and its inhibition promotes GC differentiation and follicle development by binding to *FSHR*^24^. The miR-30 family plays a critical role in cell proliferation and is upregulated in broody chicken GCs. miR-30a-5p can alleviate follicle atrophy by inhibiting autophagy and apoptosis of chicken GCs by targeting *Beclin1*^20^. miR-101-3p binds to the immune-related gene *IRF4* to decrease pro-inflammatory cytokines^41^ and potentially modulates ovarian development by targeting *BMP5*^11^. miR-449b-5p regulates steroidogenesis and estrogen secretion by targeting *IGF2BP3*^52^, and circadian miR-449c-5p targets *ATPZB4* to regulate the calcium signaling pathway^53^. The target genes of miR-7 are enriched in key reproductive pathways that may be linked to egg-laying traits, as they modulate primordial follicle activation, growth, and ovulation^54^. The miR-132 family is implicated in affecting the sexual maturity of laying hens by regulating lipid metabolism through targeting related genes^55^. miR-1662 affects egg laying by regulating metabolism and immunity^56^. miR-128 is associated with lipid regulation during follicular selection in geese^12^.

Our functional enrichment analysis identified several follicular development-related pathways targeted by multiple miRNA-mRNA-pathway networks. For instance, Oocyte meiosis and Progesterone-mediated oocyte maturation have been implicated in the regulation of follicular development and maturation^3,15^. Autophagy and Cellular senescence pathways have been associated with follicular atresia^7,15^. The TGF-beta and Wnt signaling pathways have been found to be involved in follicular selection^11,57,58^. *TGF-β* is believed to regulate the expression of *Hsd3b* during ovulation, which is crucial for progesterone (P4) synthesis. P4, derived from steroid hormones, plays a vital role in avian reproduction^57^. The GCs of the most recently ruptured follicle (POF) exhibit a robust secretory capacity for P4^7^, and POF may serve as a transient supplementary endocrine gland in the chicken ovary, stimulating the development of the pre-hierarchical follicles^59^. Due to the POFs can promote the proliferation of theca externa cells of SWF^59^, we observed an exclusive phenomenon that the number of up-regulated miRNAs are higher than down-regulated miRNAs in POF and SWF comparison. However, in our study, EMC receptor interaction, which is essential for maintaining normal ovarian function and follicle development^60^, GnRH secretion, and Cholesterol metabolism involved in follicular maturation^49,58^, and the Calcium signaling pathway implicated in ovulation^61^ were not significantly enriched.

Multiple regulatory networks exhibit crosstalk at various stages, involving the targets of gga-miR-202-5p, gga-miR-20b-5p, gga-miR-128-3p, and gga-miR-21-5p. These miRNAs are involved in pathways such as Oocyte meiosis, Progesterone-mediated oocyte maturation, Cellular senescence, Autophagy, TGF-beta signaling pathway, and cellular junction, which play roles in follicular development, maturation, selection, and atresia. miR-202-5p is crucial for follicular development and selection as it inhibits lipid metabolism and steroidogenesis^62^ and promotes apoptosis while suppressing the proliferation of geese GCs^50^. Its significant upregulation in large follicles also influences the final maturation of goat-dominant follicles^63,64^. gga-miR-20b-5p is associated with the proliferation of chicken primordial germ cells (PGCs)^51^. Moreover, it is involved in lipid metabolism, and its overexpression can alleviate adipocyte differentiation in the context of human PCOS^48,65^. gga-miR-128-3p regulates follicular selection by promoting GC apoptosis, inhibiting lipid synthesis, and reducing the secretion of progesterone and estrogen^47^. In rat GCs, miR-128-3p improves follicular development by inducing apoptosis^32^. The expression of gga-miR-21-5p increases with the follicle maturation, peaking in POF. miR-21 has been found to have anti-apoptotic effects^66^ and contributes to the involution of sheep atretic follicles^67^. It is positively associated with oocyte maturation, and the knockdown of miR-21 in GCs increases apoptosis and is associated with reduced ovulation rate^10,21–23,34^.

## Conclusions

We conducted miRNA profiling of GCs from peak laying hens across 10 grades, representing pre-hierarchical, preovulatory, and postovulatory follicles stages. Through this analysis, we identified a substantial number of DDMs and DDGs associated with follicular development, maturation, selection, and regression. These results provide a solid foundation for further investigations into the underlying mechanisms of reproductive performance, shedding light on global functional genes and essential pathways involved in domestic poultry. Additionally, these findings serve as a valuable resource for poultry breeding, aiming to enhance egg-laying capacity.

### Supplementary Information

Supplementary Information.

## Data Availability

The datasets generated for this study have been deposited in the National Center for Biotechnology Information (NCBI) with an SRA accession number PRJNA948873 (https://www.ncbi.nlm.nih.gov/bioproject/PRJNA948873/).
